# Recent Radiomics Advancements in Breast Cancer: Lessons and Pitfalls for the Next Future

**DOI:** 10.3390/curroncol28040217

**Published:** 2021-06-25

**Authors:** Filippo Pesapane, Anna Rotili, Giorgio Maria Agazzi, Francesca Botta, Sara Raimondi, Silvia Penco, Valeria Dominelli, Marta Cremonesi, Barbara Alicja Jereczek-Fossa, Gianpaolo Carrafiello, Enrico Cassano

**Affiliations:** 1 Division of Breast Radiology, IRCSS, IEO European Institute of Oncology, 20141 Milan, Italy; anna.rotili@ieo.it (A.R.); silvia.penco@ieo.it (S.P.); valeria.dominelli@ieo.it (V.D.); enrico.cassano@ieo.it (E.C.); 2 Department of Radiology, University of Brescia, Piazzale Spedali Civili 1, 25123 Brescia, Italy; giorgiomaria.agazzi@gmail.com; 3 Medical Physics Unit, IRCSS, IEO European Institute of Oncology, 20141 Milan, Italy; francesca.botta@ieo.it; 4 Molecular and Pharmaco-Epidemiology Unit, Department of Experimental Oncology, IRCSS, IEO European Institute of Oncology, 20139 Milan, Italy; sara.raimondi@ieo.it; 5 Radiation Research Unit, IRCSS, IEO European Institute of Oncology, 20141 Milan, Italy; marta.cremonesi@ieo.it; 6 Department of Oncology and Hemato-Oncology, University of Milan, 20122 Milan, Italy; barbara.jereczek@ieo.it; 7 Division of Radiotherapy, IRCCS, IEO European Institute of Oncology, 20141 Milan, Italy; 8 Radiology Unit, Foundation IRCCS Cà Granda Ospedale Maggiore Policlinico, 20122 Milan, Italy; gianpaolo.carrafiello@unimi.it; 9 Radiology Department of Health Sciences, University of Milano, 20122 Milan, Italy

**Keywords:** radiomics, breast cancer, radiology, oncology, medical physics, radiotherapy, artificial intelligence

## Abstract

Radiomics is an emerging translational field of medicine based on the extraction of high-dimensional data from radiological images, with the purpose to reach reliable models to be applied into clinical practice for the purposes of diagnosis, prognosis and evaluation of disease response to treatment. We aim to provide the basic information on radiomics to radiologists and clinicians who are focused on breast cancer care, encouraging cooperation with scientists to mine data for a better application in clinical practice. We investigate the workflow and clinical application of radiomics in breast cancer care, as well as the outlook and challenges based on recent studies. Currently, radiomics has the potential ability to distinguish between benign and malignant breast lesions, to predict breast cancer’s molecular subtypes, the response to neoadjuvant chemotherapy and the lymph node metastases. Even though radiomics has been used in tumor diagnosis and prognosis, it is still in the research phase and some challenges need to be faced to obtain a clinical translation. In this review, we discuss the current limitations and promises of radiomics for improvement in further research.

## 1. Introduction

In the last few years, the inclusion of standard digital imaging among the possible sources of big data for precision medicine has represented one of the new frontiers of research. Particularly, radiomics, the “omic” field related to diagnostic imaging, has been viewed as a great opportunity for several medical fields, yielding the most interesting results in oncology. Radiomic tumor analysis, including intra and inter-tumor heterogeneity, tumoral micro-environment and infiltrating cells, aims to extract quantitative features from medical imaging that are potentially beyond the perception of the human eye, in order to uncover novel features that are associated with treatment outcomes, disease molecular expressions or patient survival.

Moreover, recent technological advances in the field of computation and artificial intelligence (AI) applied to radiomics, hold promise in addressing challenges in its application [1,2]. In breast cancer, radiomics has been recently applied to identify molecular phenotypes and lymph node metastases, to evaluate treatment response and to predict disease survival [3,4,5,6,7,8].

## 2. Why Do We Need Radiomics in the Breast Cancer Care?

Breast cancer (BC) is the tumor with the highest incidence worldwide [9]. Although the screening and the advancements in personalized treatments have improved survival, it is estimated that BC related deaths will increase 43% globally from 2015 to 2030 [10].

At present, the diagnosis of early BC is based on radiological evaluation and histopathological confirmation of malignancy on biopsy samples [11,12]. With such approach is possible to characterize molecular alterations safely and effectively but have inherent limitations due to tumor heterogeneity and accessibility, as well as from procedure related risks [13,14].

Notably, the BC heterogeneity (which also undergoes temporal variation) is recognized as an important factor leading to cancer treatment failure and poor prognosis [15]. The quantification of heterogeneity relies on identification of various biomarkers, by the use of either tissue biopsy or medical imaging features. While the image-guided biopsies offer excellent spatial resolution for tissue analysis on a cellular scale and allow genetic and molecular sequencing [16]; however, biopsy is limited by risks of invasive procedures and focal sampling errors and limitation by tumoral characteristics such as small size, location or heterogenous necrosis [3,13,14].

Radiomics has the capability to analyze both temporal and spatial heterogeneities through quantitative serial data evaluation and recent advancements in radiomics analysis provide the potential to retrieve useful incremental information to characterize molecular alterations from standard imaging data in a non-invasive way [16]. However, radiomics currently suffers from lack of validation and standardization: further developments and improvements are needed to achieve reliable and clinically applicable results [14,16,17].

Whatever the method, the accurate biological assessment of BC is crucial because each subtype has its own biological and genetic profile with a subsequent different prognosis and treatment options. Subtypes are characterized by distinct molecular profiles, proliferation rates, tumor receptors and grade. Due to their potential effect on prognosis and clinical management, four biomarkers are tested consistently in biopsies and excision specimens of BC: estrogen receptor (ER), progesterone receptor (PR), human epidermal growth factor receptor 2 (HER2) and Ki67 antigen [11].

Prognosis for early-stage ER-positive/HER2-negative BC is usually excellent [18] and most of the invasive BCs hormone receptor positive show a more indolent clinical course [19]. Therefore, PR and ER status are considered as strong positive predictive factors since the advent of targeted hormone therapy.

At present, tumors are often classified as “luminal-A” or “luminal-B”, human epidermal growth factor receptor 2 (HER2)-overexpressing and triple negative (TN), based on immunohistochemical analyses [20,21]. Luminal A are cancers with ER+, PR+ and Ki67 < 20% and ER+, PgR+/− and Ki67 < 14%, and the best prognosis. Luminal B are cancers with ER+ but may have variable degrees of ER/PR expression, are higher grade and have higher proliferative fraction. HER2-overexpressing BC are ER−, PR− and HER2+ and they have a poorer prognosis than luminal BCs while the TN cancers (ER−, PR− and HER2−) have the poorest survival rate. These classifications could be used to inform adjuvant treatment decisions. Specifically, either grading or Ki-67 could be used to distinguish between the Luminal-A- and B-like [21].

Accordingly, biomarkers are crucial to tailor treatment strategies to the individual patient in the paradigm of personalized medicine [22]. However, the only way to obtain the biological profile of BC is currently through a tissue sample via surgery or biopsy. For this purpose, a new non-invasive technique based on imaging would be worthwhile. Radiomics, through the conversion of standard digital imaging into mineable, quantitative data expressing different tumor properties, has gained recognition as a new tool in the field of cancer care for non-invasively profiling of BC [1,3,7,8].

Particularly, the ultimate purpose of radiomics applied in BC care should be early diagnosis of BC and prediction of its clinical course and biological aggressiveness in order to optimize treatment [23].

The imaging evaluation of BC through mammography, ultrasound (US) or magnetic resonance imaging (MRI) is currently essentially qualitative. This includes subjective evaluations such as tumor morphology/structure, type of enhancement, anatomic relationship to the surrounding tissues. However, to reach a truly personalized medicine, a quantitative evaluation is demanded too [3]. Data derived from radiomics investigation, such as the intensity, shape, textural related features and wavelength related transforms, may provide valuable information to differentiate benign from malignant lesions, to predict treatment response, to assess cancer molecular profile and to derive robust models that combine multidisciplinary information [24,25,26,27,28,29,30,31].

## 3. The Workflow of a Radiomic Study

Most of radiomics studies concerns its application in the oncological field and the first step is generally to acquire the appropriate images. The Quantitative Imaging Biomarker Alliance and Quantitative Imaging Network have defined standardized imaging protocols and recommendations in the field of quantitative imaging [32] to improve the reproducibility of radiomics studies, which remains one of the biggest drawbacks currently limiting their clinical application.

Radiomics features are generally extracted from routine medical images that decode information about a region of interest (ROI) which are specified to limit the spatial extents of the analysis and can be delineated manually, semi-automatically or automatically, with increased reproducibility for textural features extracted with automatic segmentation algorithms compared to free-hand region delineation [33]. Feature extraction from the ROIs is performed using specific algorithms and are thus objective imaging features, with standard mathematical definition of the most common features [17].

**Figure 1:**
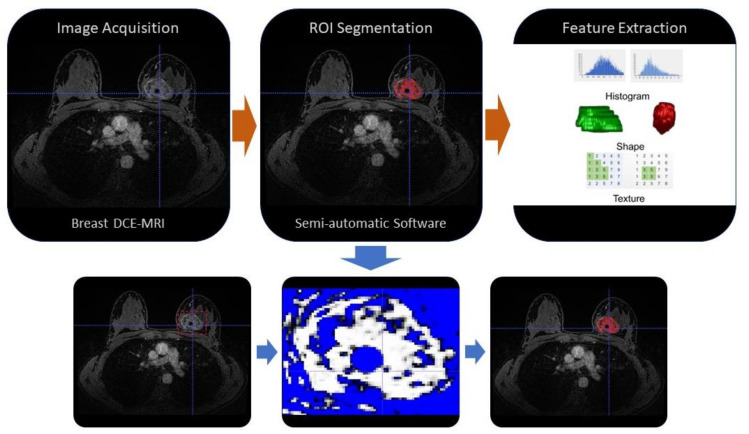
Example of MRI-based radiomics workflow. The first phase is the image acquisition (i.e., by breast MRI with contrast-enhancement sequences), then (orange arrow) the ROI segmentation could be performed manually or by automatic or semi-automatic software, finally (orange arrow) the radiomic features are extracted and selected by algorithms. An example of a semi-automatic segmentation by a threshold value method is shown in the three figures below (blue arrows). ROI: region of interest, DCE-MRI: Dynamic contrast enhancement-Magnetic resonance imaging.

An example of MRI-based radiomics workflow for features extraction is shown in Figure 1.

The features can be broadly classified into four categories: morphological, histogram-based, textural and related to the gray level co-occurrence matrix and to transform-based features [32]. Morphological features describe different aspects of the lesion shape, such as volume, surface area, convexity or the borders heterogeneity. Histogram-based features characterize the histogram of voxel intensities, including the average value, standard deviation and parameters related to the histogram shape such as skewness and kurtosis. Textural features focus on the spatial arrangement of voxel intensities, trying to capture different properties of their distribution in terms of heterogeneity, randomness, presence of clusters or privileged signal directions. All these features can be calculated from the images as they are, or after applying mathematical transforms, such as wavelet of Laplacian of Gaussian (LoG), resulting in the so-called transform-based features. While hundreds or thousands of features may be computed, only a selection of fewer (and more specific) features is required to compute a clinically useful radiomic signature. Features whose value is not stable when images are repeatedly acquired under the same experimental condition (referred to as unstable or not repeatable features) should be identified a priori, by means of phantom studies or, if feasible, test-retest acquisitions in the clinical setting and eliminated [34]. Usually, a big gap between the number of features extracted (p) within a study and the number of patients actually recruited (n) remains, leading commonly to p>>n, with the risk to build radiomic models with high predictive accuracy in the experimental dataset but with extremely poor generalizability of the results, due to precise modelling of dataset “noise” instead of the true biological behavior. To overcome this problem feature selection and dimension reduction is of utmost importance, and different approaches can be performed, including rigorous algorithms such as principal component analysis, LASSO or Boruta [32]. The desired response variable differs based on the study, and models are built using the selected features to suit specific aims. For classification problems (e.g., benign vs malignant lesions), various classifiers are used including support vector machine (SVM), random forest (RF) and XGBoost classifiers. To predict continuous variables, such as the expression of biological markers, various regression methods including linear regression, regularized linear regression and RF are commonly used. For prediction of survival, Cox regression models with or without LASSO approach are finally performed.

Most radiomics studies involve a mixture of biomedical imaging specific techniques related to signal processing and proper AI applications, a broad field of computational techniques which includes machine learning (ML) and deep learning (DL) algorithms, the latter being often “black-box” and self-learning neural networks, with less dependence on human input in the model building step [33]. Given the high number of features obtained within radiomics studies and the often-non-linear relationships involved, these techniques offer a better approach in clinical predictive modeling compared to traditional inferential statistic and if properly applied, can limit model overfitting. Since a number of radiomics studies focused on BC are limited to single-center data lacking external validation, cross-validation with a leave-one-out, k-fold approach or with bootstrapping can be adopted using splits of the data into training and validation sets [33].

However, the optimal method of validation remains external dataset independent validation, which is typically accomplished in multi-center studies. However, acquiring multi-center data is challenging, so the solution may be to leverage an open database such as the cancer genome atlas program (TGCA), to acquire the external validation data.

As previously elucidated, reproducibility and standardization of radiomics analysis is currently the biggest issue. This partly because of the intrinsic high number of different steps involved and partly because every one of each can be performed in several different ways. The retrospective nature of studies, the heterogeneity of software and the variability of the radiomics features that can be extracted in the different studies raise legitimate concerns regarding the potential lack of reproducibility in radiomics. It is good practice to acquire imaging data using standardized settings that should be well documented in published papers, in order to be accurately evaluated during peer-review and be available to different research teams working on the same field. Data obtained under such settings should be shared on public repositories in order to receive appropriate external validation.

## 4. Radiomics Application in Breast Cancer

Even though there are studies about radiomics based on mammography, digital breast tomosynthesis (DBT), US and even PET/CT, in BC imaging scenario the radiomics approaches have been investigated mainly with MRI and, in the very last few years, with the contrast enhancement spectral mammography (CESM). However, results of most studies have been derived from relatively pure study designs, with homogeneous patient populations where the MRI was sourced from specific scanner systems and a single field strength. This limits their wider applicability and generalizability at present.

Table 1 and Table 2 summarize in our opinion the most relevant original studies and reviews, respectively, on radiomics in breast imaging published in peer reviewed journals from 01/2018 to 01/2021. Results from other interesting studies are briefly discussed only in the text.

In the following sections, studies on the current main applications of radiomics in BC care are discussed.

## 5. Discrimination between Benign and Malignant Breast Lesions

The early identification and characterization of BC is essential to improve outcomes in patients because small non-metastatic disease can be effectively treated with curative intent [11,52,53].

To detect a malignant breast lesion, dynamic contrast-enhancement MRI (DCE-MRI) is currently the imaging technique with the best accuracy performance [54]. Accordingly, most radiomics studies are based on such technique. A recent DCE-MRI-based radiomics study by Zhou et al. [41] (Table 1) used 99 texture and histogram parameters from 133 patients to differentiate between benign and malignant lesions. Their model resulted in an accuracy of 91% when using the smallest bounding box of peritumoral tissues in segmentation, showing that including proximal peritumor tissue provided higher accuracy than including in segmentation tumor alone or larger boxes. Other MRI-based studies considered both DCE and diffusion weighted imaging (DWI), such as the one by Xie et al. [43] (Table 1). They recently analyzed features extracted from images of 134 invasive ductal cancers, founding highest accuracy of 91% for comparing triple negative to non-TN cancers. Previously, Jiang et al. [55] already showed how combining DCE-MRI and DWI with ADC values increased the overall accuracy for discriminating of malignant and benign breast nodules to 0.90. In 2018, a retrospective study [56] examined unenhanced DWI-based radiomics to predict the malignant nature of suspicious breast lesions detected on screening mammography, showing that lesions classified as BI-RADS 4 or 5 by mammography resulted in 70% of false-positive findings while retaining 98% of sensitivity.

Using mammography, Li et al. [57] analyzed the radiomic features of the breast with a suspicious lesion alongside with contralateral health breast in 182 patients (106 malignant and 76 benign), showing that the combined lesion and parenchyma classifier in the differentiation of malignant and benign mammographic lesions was better than using the lesion features alone. In 2019, a sub-study of a multi-center and prospective study leaded by Tagliafico et al. [58] applied a radiomics approach to Digital Breast Tomosynthesis for the first time to differentiate normal from malignant breast tissue in patients with dense breasts in a small number of 40 patients, showing encouraging results.

Recently, Massafra et al. [59] used CESM to discriminate benign and malignant breast lesions based on radiomic analysis of 53 patients with BC resulting with the aid of the random forest classifier in the best prediction of benign/malignant with median values for sensitivity and specificity of 88.37% and 100%, respectively.

Finally, there are examples of US-based radiomics for discrimination of malignant breast lesions, such as the recent study by Luo et al. [60] based on 315 BCs patients which showed that nomograms combining the radiomics score and BI-RADS category improved the discrimination of benign and malignant lesions than either the single radiomics score or the BI-RADS category.

## 6. Prediction of Breast Cancer’s Molecular Subtypes

Once a breast lesion is diagnosed as malignant, its molecular subtype has to be assessed. In 2017, Fan et al. [61] analyzed a combined model of DCE-MRI-based radiomics features and clinical information to predict luminal A, luminal B, HER2-overexpressing and TN. The AUC values were 0.87, 0.79, 0.89 and 0.92, respectively.

Most of the radiomics studies in this field are based on MRI. In 2019, Xie et al. [62] used texture features extracted from a quantitative ADC map and DCE to detect TNBC with an AUC of 0.71 (TNBC vs luminal A), 0.76 (TNBC vs HER2 positive) and 0.68 (TNBC vs non-TNBC). In 2020, Demircioglu et al. [39] (Table 1) showed the usability of a simplified and rapid approach to tumor for MRI-based tumor decoding and phenotyping of BC on a population of 98 patients. Notably, they evaluated the molecular subtype, hormonal receptor status, Ki67- and HER2-expression, ALN metastasis as well as grading considering 13.118 radiomic features extracted with a VOI-based approach. Involvement of the ALN could be predicted with an AUC of 0.80, while ALN metastasis yielded an AUC of 0.71. Receptor status predictions yielded AUCs of 0.67–0.69, Ki67 0.81 and HER2 Expressions 0.62, which are promising results but not enough to be applied in clinical practice as a substitute of tissue samples. In 2018, Liang et al. [44] (Table 1) proposed a noninvasive Ki67 predictor status based on breast radiomics features extracted from 318 breast MRI. Their customized radiomic score based on T2WI was significantly associated with the Ki67 status, suggesting a new radiomics marker might pre-operatively predict Ki67 expression in patients with BC.

In addition to MRI, even CESM was used to extract radiomics features for prediction histological outcome, HER2-positive and TN BCs from 52 patients (68 lesions) with encouraging results [63]. Specifically, the highest performances were obtained for discriminating HER2+/HER2− (90.87%), ER+/ER− (83.79%) and Ki67+/Ki67− (84.80%).

## 7. Prediction of Response to Neoadjuvant Chemotherapy

In the last decade, neoadjuvant chemotherapy (NACT) has been increasingly used in the treatment of operable BC and it is associated with a positive response, especially in women with ER-negative BC and it decrease the rate of recurrence and of BC mortality [64].

The achievement of pathological complete response (pCR) is a powerful prognostic factor for long-term outcome, and it is considered as the only currently validated biomarker of survival, but it can only be assessed at surgery [10,65]. Therefore, radiomics may allow a non-invasive and earlier detection of resistance to treatment to avoid in some patients (namely, the non-responders to NACT) the unnecessary toxicity and delays access to other potentially effective therapies. At the same time, the neoadjuvant setting provides a unique opportunity for in vivo assessment of tumor response, evaluation of biological markers of responsiveness or resistance and to study intermediate endpoints indeed [66].

Previous studies already proposed prediction models of pCR to NACT in BC based on MRI [3,10,67,68,69], and some authors suggested the possibility to predict the pCR by extracting radiomics features from pre-NACT breast MRI, obtaining statistically significant results [5,22,70,71,72].

In 2020, Choudhery et al. [5] (Table 1) used morphological and 3D textural features to predict the molecular subtype and the pCR in 259 BC women underwent with NACT. Significant differences in minimum signal intensity and entropy were found among the tumor subtypes. Sphericity in HER2+ BCs and entropy in luminal BCs were significantly associated with pCR. Multiple features demonstrated significant association with pCR and residual tumour burden in TNBC with SD of intensity achieving the highest AUC for pCR in TN BCs.

In 2017, Braman et al. [71] evaluated radiomic features based of both peri- and intra-tumoral regions on pre-treatment DCE-MRI to predict the pCR to NACT in 117 BC patients. Their results showed that peri-tumoral radiomics contributed to the prediction of the pCR of HER2+ BC patients, yielding a maximum AUC of 0.74 within the testing set.

Previously, other authors [22,61,70,72,73] showed that quantitative analyses of radiomic features (morphologic, texture and dynamic features) from pretreatment breast DCE-MRI data in BC patients could be used as valuable image markers that are associated with pCR to NACT. In the above-mentioned studies, DCE had been used more frequently than DWI to extract radiomics features as it can provide the kinetic characteristics of the contrast agent by producing pharmacokinetic maps indeed.

In a multicenter study, Liu et al. [67] utilized multiple MRI sequences, including DWI, to predict pCR to NACT in patients with BC. In 586 patients radiomic score was calculated using 13,950 features from MRI quantitatively, providing a promising tool for predicting response of patients with advanced BC and showing a potential and practical value in clinical practice.

Parikh et al. [74] used unenhanced MRI data evaluating whether changes in MRI textural features could predict the pCR in a small number of patients with BC who underwent NACT. Using histogram-based features, they showed how an increase in T2WI uniformity and a decrease in T2WI entropy after NACT could predict pCR as compared to BC size change.

In addition to MRI, Wang et al. [75] recently developed and validated a CESM-based radiomics nomogram to predict NACT-insensitive BC prior to treatment. In 117 patients, their radiomics nomogram that incorporates 11 radiomics features and 3 independent clinical risk factors, including Ki-67 index, background parenchymal enhancement (BPE) and HER-2 status, showed an encouraging discrimination power with AUCs of 0.877 (95% CI 0.816 to 0.924) and 0.81 (95% CI 0.575 to 0.948) in the training and validation sets, respectively.

## 8. Prediction of Lymph Node Metastases

Involvement of ALN is an independent predictor for disease outcomes in patients with BC [76,77,78]. At present, definitive diagnosis is reliant on pathological examination by invasive lymph node tissue sampling from surgery or biopsy. This is because imaging assessment based on nodal size measurement and/or morphological criteria has limited accuracy, and apical nodes in the axilla are poorly visualized by US at the time of diagnosis [79,80].

In the published literature to date [6,76,77,78,80,81,82,83,84,85] radiomics has been found to have moderate to good diagnostic accuracy for the determining the nodal status (AUC 0.60–0.90) in patients with BC.

In some studies [6,81,82,83], the prediction using a combination of radiomics features and clinical risk factors led to further improvement in the identification of nodal status. Particularly, Dong et al. [82] showed how radiomics features extracted from DWI sequences were highly correlated with ALN metastases than those extracted from ADC. Moreover, some studies have investigated radiomics nomograms based on mammography [86], CESM [36], US [87] and even CT [88] to predict axillary lymph node metastases pre-operatively. Once again, authors built the radiomic score from a huge number of radiomic features and then incorporated additional radiological and clinicopathological findings.

## 9. What Next?

As more research is conducted, the body of published literature is rapidly growing. In the next future radiomics may become the standard in MRI-based tumor assessment, with AI algorithms totally skilled of carrying out complex data analysis under the precise guidance of radiologists.

Currently, radiomics is an appealing technique in research but it has not been fully applied yet in the clinical setting but multidisciplinary and translational studies are still required, gather the amount of data needed to implement radiomics on a wide scale. As it is difficult to acquire consistent imaging and acquire uniform results that can be applied in clinical practice [89] (due to imaging acquisition from different machines, varied technical parameters and slice thickness and diverse reconstruction algorithms), some techniques to deal with multicentric data have been proposed such as ComBat [90]. However, radiomics models based on transparency of methodologies and developed using standardized acquisition techniques and high-quality data can overcome confounders arising from differences among centers’ workflows. Finally, the sample size of radiomics analyses is another crucial issue in predictive models: larger samples can increase prognostic accuracy and, at the same time, can make it possible to use AI techniques and DL algorithms with more robust results. However, the samples of the above-mentioned studies are not big enough, and these models should be validated in further research.

## 10. Role of Artificial Intelligence and Big Data in Radiomics

The possibilities of using texture analysis and other advanced approaches such as ML and DL in radiomics are wide open as radiomics studies have numerous degrees of freedom and need large datasets [91,92,93,94].

At present, the best method to analyze big data is based on AI technology, which consists of flexible mathematical models that use algorithms to identify complex nonlinear relationships within such data: ML is a subfield of AI which allows machines to learn without being specifically programmed, and it has been applied in radiomics [95,96]. Among the techniques that fall under the ML umbrella, DL has emerged as one of the most promising [97]. This essentially because DL allows a continuous improvement towards a better performance with lower error rate while ML reaches an error rate that cannot be further lowered adding other data to the process [98]. Moreover, while in ML handcrafted features are pre-defined using domain expertise, in DL the algorithm is able to learn specific features from the data themselves [33]. Accordingly, there is no need to specify pre-defined features as the same algorithm can solve many different tasks. On the other hand, DL till needs time before playing a significant practical role in radiomics, especially applied cancer research, due to the limitations of the available big-data which usually still lack complete characterization of the patients and poor integration of individual datasets [99]. Finally, AI suffers from the interpretability issue that may represent a main challenge for researchers who want to understand how certainly studies comes to conclusions, which features have been selected, and so how to recognize (and interpret) possible failures. This also has a practical drawback when communicating the results to the clinician, which may not be able to understand all the processes behind the DL proposed clinical response.

However, AI already showed its utility in radiomics studies. Nie et al. [100] generated higher-quality images adopting DL-based image synthesis to match different imaging settings. Havaei et al. [101] used a DL segmentation algorithm to automatically perform difficult segmentation tasks and their good results suggest the possibility to eliminate the need for manual segmentation. Finally, DL could help in the feature extraction step providing insights for new features as it may be capable of learning relevant features from the data themselves [33].

## 11. Standardization and Curation of Radiomics Data

Since for developing a radiomic workflow enforced by AI is required a big training dataset [102], radiology is in well-poised to benefit from it thanks to its abundance of data. Nevertheless, irregular completeness and different quality of data entry, as well as the interoperability between different providers, are still a non-negligible obstacle for the further development of radiomics in clinical practice. To appropriately train AI algorithms, the abundance of data that are acquired with radiological exams might be not enough indeed because most of the health-related data are unstructured and not standardized yet [98,103]. Imaging data is far from standardized as different hospitals use different systems to acquire and to store them, and it can be incomparable due to the fast development of new equipment/device/system and to the differences in the technical implementation used by the disparate vendors. In case of multicenter collaboration [104,105], the situation could be even more difficult as radiomic data are more heterogeneous and variable [106]. In this regard, standardization is the process of transforming data into a common format which can be understood and shared across different tools and methodologies. This is a crucial point in radiomic studies. The first step is the standardization of techniques [107,108]: in radiology, for instance, the size of a mass in organs can be compared consistently if the comparison are performed with exactly the same imaging protocol [104]. In addition, segmentation of an index lesion in the training process of an AI system requires the most uniform images possible. Unfortunately, o widely accepted standard o store and communicate segmentation results between different tools from different vendors/sources is still commonly used [1,109,110]. Two main example of high quality standardized annotation methods, namely the Annotation and Image Markup standard and the DICOM Presentation State [111,112], are still little used by software developers to report on annotations indeed [113]. Without early efforts to optimize interoperability, the practical effectiveness of AI in radiomics will be severely narrowed. Therefore, a set of standards would be necessary to allow integration between these different algorithms and to allow AI techniques to be used in different centers, from different users, on different equipment.

The collection and sharing of heterogeneous (from the imaging point of view) dataset, properly cured and homogeneous from a clinical standpoint, can be extremely useful to train and test feature selection and harmonization methods, aiming at identifying robust procedures and/or features, able to guarantee generalizability. This is especially relevant when ML techniques are used to analyze handcrafted radiomic data extracted from images, an approach which should be preferable when the number of available data is not high enough to allow the use of DL methodology. Conversely, in presence of sufficiently wide datasets, DL methodology might be favored and image dataset heterogeneity would represent an advantage more than an issue, DL architecture having the potential to incorporate in the deep layers of the network image processing and features selection tasks.

Broadly speaking, the lack of appropriate big datasets is a key obstacle to a large introduction of AI systems in healthcare [94,96,114], and the needed level of data curation is strictly related to the number of available cases and to the AI methodology implemented, and the other way around. Scientists and researchers have the fundamental role of choosing the best methodology in relation to the dataset characteristics, and to collect adequate datasets for the aim of the study, also in relation to the methodology they plan to implement.

The collection of external, independent dataset for validation is fundamental for testing the performance of predictive models in a validation setting. The standards for prediction model reporting which are currently used need to be updated. Radiomics datasets for AI algorithm training, testing and validation should be updated and developed including statistical metrics for validation, parameters for clinical integration and pathways for assessing algorithm performance in research and even in clinical practice [106,115].

As standardized evaluation of the performance, reproducibility and clinical utility of radiomics findings is needed. The radiomics quality score is a specific system of metrics with the purpose to determine the validity and completeness of radiomics studies [116,117], similarly to the Transparent Reporting of a multivariable prediction model for Individual Prognosis Or Diagnosis (TRIPOD) initiative [13]. These indications are applied to a radiomics-specific design that considers high-dimensional data and modeling, and highlights the clinical adoption of modeling research as in the TRIPOD guidelines [118].

## 12. Radiomics Data Sharing

Since radiomic data are needed for validation and multi-centers cooperation, they may need to be shared across multiple institutions and across nations for a widespread implementation. Accordingly, there is the need to compliance with regulatory frameworks when using personal data such as health information [1]. Data would need to be anonymized as the rules of patient privacy and the cybersecurity measures will be increasingly important in radiomic research [1,119,120].

In EU, regulators recently updated the legislation concerning data protection and cybersecurity with GDPR [99,121]. Furthermore, with the Cybersecurity Directive [122], they set out a number of requirements for EU Member States to prevent cyberattacks and, eventually, keep consequences under control [122]. In the US, the Health Insurance Portability and Accountability Act is a compliance focus for what concerns health information [123] defining standards and safeguards that protect confidential data and personal health information that apply to all healthcare providers, insurers and other healthcare entities.

On the other hand, the current healthcare environment still holds little incentive for data sharing [124]. Some policymakers have proposed creating anonymized benchmarking datasets including a local calibration, which is crucial because radiomics features may have local or cultural-specific parameters that may not be generalizable to different populations.

Concerning the radiomics studies in the field of BC, the Cancer Imaging Archive [125] is a good example of a functional service which hosts a large archive of anonymized medical images of tumors with related data (e.g., patient outcomes, treatment details, genomics, pathology, expert analyses). Such archive is accessible for public download.

Other example of data-sharing efforts include biobanks and international consortia for medical imaging databases, such as the Cardiac Atlas Project [126], the Visual Concept Extraction Challenge in Radiology Project [127], the UK Biobank [128] and the Kaggle Data Science Bowl [129].

Finally, the other milestone in radiomics will be transparency. The accuracy of radiomics performance relies massively on the quality of the inputted data: accordingly, poorly labelled data will yield poor results [130] and transparency of labelling allows that others can critically evaluate the radiomic workflow process.

## 13. Conclusions

The recent studies discussed in this review show that radiomics applied in BC is an expanding and promising research topic. However, the application of radiomics in clinical practice is still hampered by some pitfalls. Lessons from recent experience, further advances in technology (including development of AI) and efforts in curation and standardization of data and methodologies among researchers would make radiomics a more robust and trustable field in both research and clinic in BC care.

## Figures and Tables

**Table 1:** Original studies on radiomics in breast imaging published in peer reviewed journals from 01/2018 to 01/2021, classified on modality/technique and ordered by newest first. Relevant papers were obtained with a scoping review approach, using the following set of keywords and the relative controlled vocabulary terms (Mesh/Emtree): (radiomic* OR textur*) AND (breast) AND (cancer* OR malign* OR neoplas* OR metast* OR tumor* OR tumour*). The same approach was conduct for Table 2, which includes only review papers. ALN: axillary lymph node, AUC: area under the curve, BC: breast cancer, CESM: contrast enhancement spectral mammography, DCE: dynamic contrast-enhanced, ML: machine learning, NACT: Neoadjuvant Chemotherapy, pCR: pathological complete response, SD: standard deviation, TNBC: triple-negative breast cancer, T1WI: T1 weighted imaging, T2WI: T2 weighted imaging, VOI: volume of interest.

Modality/Technique	Author	Purpose	Radiomics Features Category and Purpose	Population	Results	Conclusion
CESM	Lin et al., 2020 [35]	Identification of benign and malignant BC lesions <1 cm	Radiomics features extracted from low-energy and recombined images on CC position	139 patients	The radiomics nomogram combined with Radiomic-score, BI-RADS category and age showed AUC of 0.940.	The radiomics nomogram incorporated with CESM-based radiomics features, BI-RADS category and age could identify benign and malignant BC <1 cm
CESM	Mao et al. 2020 [36]	Pre-operative prediction of ALN metastasis	LASSO logistic regression was established for feature selection and utilized to construct radiomics signature	394 patients	ROC curves of 0.774, 0.767 and 0.79 in the training, internal validation and external validation sets, respectively.	Authors identified the cutoff score in the radiomics nomogram as −1.49, which corresponded to a total point of 49 that could diagnose ALN metastasis with a sensitivity of >95%.
MRI	Tan et al., 2020 [6]	Value of radiomics feature extracted on the fat-suppressed T2WI for preoperative predicting ALN metastasis in BC	17 texture features, 5 first-order statistical features, patient age, tumor size, HER2 status and thrombus	329 BCs	Sensitivity, specificity, accuracy and are under the curve value of radiomics signature 65.22%, 81.08%, 75.00% and 0.819.	The MRI-based radiomics signature and nomogram could be used as a non-invasive and reliable tool in predicting ALN metastasis.
	Choudhery et al., 2020 [5]	Assessment of BC molecular subtype, pCR and Residual Cancer Burden in BC Patients Treated with NACT	Morphological and three-dimensional MRI textural features were computed, including unfiltered and filtered image data, with different spatial scaling factors	259 BCs	Differences in minimum signal intensity and entropy among the tumor subtypes were significant. Sphericity in HER2+ tumors and entropy in luminal tumors were significantly associated with pCR. Multiple features demonstrated significant association with pathological complete response and residual cancer burden in TNBC with SD of intensity achieving the highest AUC for pCR in TNBC.	MRI radiomics features are associated with different molecular subtypes of breast cancer, pathological complete response and residual cancer burden.
	Hao et al., 2020 [37]	Contralateral BI-RADS 4 lesion assessment	1046 radiomic features	178 BCs	DCE-T1WI and T2WI imaging features signatures yielded an AUC of 0.77, which was better than the AUC of each signature alone.	The MRI radiomics-based ML model based on T2WI and DCE-T1WI features provided complementary information in discriminating benign and malignant contralateral BI-RADS 4 lesions.
	Lo Gullo et al., 2020 [38]	Assessment of sub-centimetric breast masses in BRCA patients	Radiomics features calculated using open-source CERR software	96 BRCA carrier	The ML model combining 5 parameters including clinical factors, GLCM-based correlation from the pre-DCE phases and first-order coefficient of variation from the 1st post-DCE phase, achieved a diagnostic accuracy of 81.5%.	Radiomics analysis improved diagnostic accuracy compared with qualitative morphological assessment alone.
	Demircioglu et al., 2020 [39]	Molecular subtype, hormonal receptor status, Ki67- and HER2-expression, metastasis of lymph nodes and lymph vessel involvement as well as grading	13.118 radiomic features extracted with a VOI-based approach	98 BCs	PR and ER status predictions yielded AUCs of 0.67–0.69, Ki67 0.81 and HER2 Expressions 0.62. Involvement of the ALN could be predicted with an AUC of 0.80, while lymph node metastasis yielded an AUC of 0.71.	A rapid approach to VOI-based tumor-annotations for radiomics provides consisternt results to other studies in the same field.
	Zhang et al., 2020 [40]	Differentiation between benign and malignant lesions	Radiomics features extracted from T2WI, T1WI, DKI, ADC maps and DCE pharmacokinetic parameter maps	207 BCs	The AUC of the optimal radiomics model, including T2 WI, DKI and quantitative DCE-MRI parameter maps was 0.921, with an accuracy of 0.833.	The model based on radiomics features from T2WI, DKI and quantitative DCE parameter maps has a high discriminatory ability for benign and malignant BC lesions.
	Zhou et al., 2020 [41]	Differentiation between benign and malignant BC lesions	99 texture and histogram parameters	133 patients	The highest accuracy of 91% was achieved when using the smallest bounding box of peritumoral tissues in segmentation.	Using the smallest bounding box containing proximal peritumor tissue as input had higher accuracy compared to using tumor alone or larger boxes.
	Liu et al., 2019 [42]	Assess lymphovascular invasion status	Radiomic signature composed of two features	149 BCs	The value of AUC for a model combining both radiomic signature and ALN status (0.763) was higher than that for MRI ALN status alone and similar to that for the radiomics signature.	The DCE-MRI-based radiomics signature in combination with ALN status was effective in predicting the lymph and vascular invasion status of patients with BC before surgery.
	Xie et al., 2019 [43]	Subtype classification of breast cancer	2498 features extracted from the DCE and DWI, together with DCE images, changing over 6 time points and DWI images changing over 3 b-values	134 invasive ductal carcinoma	Highest accuracy of 91% for comparing triple negative to non-triple negative cancers.	Whole-tumor radiomics on MRI provides a non-invasive approach for BC subtype classification.
	Liang et al., 2018 [44]	Preoperative Ki-67 status	Radiomic features based on T2W and DCE-T1WI	318 BC	The T2W image-based radiomics classifier showed significant discrimination for Ki-67 status, with AUC of 0.74 in the validation dataset.	The T2WI-based radiomics classifier was a significant predictor of Ki-67 status in patients with breast cancer while DCE-T1WI radiomic features were not able to discriminate Ki-67 status in the validation dataset.
Digital mammography	Tan et al., 2020 [45]	Pre-operative prediction of ALN metastasis	Radiomic signature nomogram combined with receptor status and molecular subtype	216 BCs	The radiomics nomogram, comprising PR status, molecular subtype and radiomics signature, showed excellent calibration and better performance for the metastatic ALN detection (AUC 0.883 and 0.863 in the primary and validation cohorts), better than each independent clinical feature and radiomics signature.	The mammography-based radiomics nomogram could be used as a non-invasive and reliable tool in predicting ALN metastasis.
Digital Mammography	Stelzer et al., 2020 [46]	Distinguish malignant from benign classification	249 image features from gray-value histogram, co-occurrence and run-length matrices	226 patients	A high sensitivity threshold criterion was identified in the training dataset and successfully applied to the testing dataset, demonstrating the potential to avoid 37.1-45.7 % of unnecessary biopsies at the cost of one false-negative.	Combined texture analysis and ML could be used for risk stratification in suspicious mammographic calcifications.
	Zhou et al., 2019 [47]	HER-2 status	186 radiomic features	306 l BCs	In the testing set the AUC of the radiomic model in assessing HER-2 status was 0.787.	Radiomics features could help in the preoperative evaluation of HER-2 status in patients with BC.
	Lei et al., 2019 [48]	Prediction of benign BI-RADS 4 calcifications	8286 radiomic features extracted from the craniocaudal and mediolateral oblique scans	212 calcifications	Six radiomic features and the menopausal state included in a radiomic nomogram could discriminate benign from malignant calcifications with an AUC of 0.80 in the validation cohort.	The mammography-based radiomic nomogram is a potential tool to distinguish benign calcifications from malignant calcifications.
PET/CT	Ou et al., 2020 [49]	Differentiating breast carcinoma from breast lymphoma	Radiomic features extracted with a local software	44 BCs	AUCs of 0.867 and 0.806 for PET radiomic and clinical model, AUCs of 0.891 and 0.759 for CT based radiomic model on training and validation data.	Models based on clinical, and radiomic features of 18 F-FDG PET/CT images could accurately discriminate BC from breast lymphoma.

**Table 2:** Review studies on radiomics in breast imaging published in peer reviewed journals from 01/2018 to 01/2021, ordered by newest first.

Reference	Modality/Techique	Purpose	Radiomics Features Category and Purpose	Population	Results	Conclusion
Reig et al., 2020 [50]	MRI	Review focused on machine learning techniques in breast MRI	Pre-processing, neural networks, deep learning, machine learning, segmentation, texture analysis	Breast malignant and benign pathology.		The Author discuss the possible future directions of machine learning in the current workflow of breast lesions assessed with MRI.
Granzier et al., 2019 [51]	MRI	Systematic review, response prediction of neoadjuvant therapy	Various radiomic feature models, evaluated with the Radiomics Quality Score (RQS)	Studies ranging between 35-414 BC	AUC values ranged from 0.83 to 0.85. The best performing multivariate prediction model, based on logistic regression analysis, showed AUC of 0.94.	The systematic review revealed large heterogeneity for each step of the MRI-based radiomics workflow. Consequently, the results are difficult to compare.

## References

[1] Pesapane F., Codari M., Sardanelli F. (2018). Artificial intelligence in medical imaging: Threat or opportunity? Radiologists again at the forefront of innovation in medicine. Eur. Radiol. Exp..

[2] Becker A.S., Marcon M., Ghafoor S., Wurnig M.C., Frauenfelder T., Boss A. (2017). Deep Learning in Mammography: Diagnostic Accuracy of a Multipurpose Image Analysis Software in the Detection of Breast Cancer. Investig. Radiol..

[3] Pesapane F., Suter M.B., Rotili A., Penco S., Nigro O., Cremonesi M., Bellomi M., Jereczek-Fossa B.A., Pinotti G., Cassano E. (2020). Will traditional biopsy be substituted by radiomics and liquid biopsy for breast cancer diagnosis and characterisation?. Med. Oncol..

[4] Zhuang X., Chen C., Liu Z., Zhang L., Zhou X., Cheng M., Ji F., Zhu T., Lei C., Zhang J. (2020). Multiparametric MRI-based radiomics analysis for the prediction of breast tumor regression patterns after neoadjuvant chemotherapy. Transl. Oncol..

[5] Choudhery S., Gomez-Cardona D., Favazza C.P., Hoskin T.L., Haddad T.C., Goetz M.P., Boughey J.C. (2020). MRI Radiomics for Assessment of Molecular Subtype, Pathological Complete Response, and Residual Cancer Burden in Breast Cancer Patients Treated With Neoadjuvant Chemotherapy. Acad. Radiol..

[6] Tan H., Gan F., Wu Y., Zhou J., Tian J., Lin Y., Wang M. (2020). Preoperative Prediction of Axillary Lymph Node Metastasis in Breast Carcinoma Using Radiomics Features Based on the Fat-Suppressed T2 Sequence. Acad. Radiol..

[7] Tagliafico A.S., Piana M., Schenone D., Lai R., Massone A.M., Houssami N. (2019). Overview of radiomics in breast cancer diagnosis and prognostication. Breast.

[8] Pinker K., Chin J., Melsaether A.N., Morris E.A., Moy L. (2018). Precision Medicine and Radiogenomics in Breast Cancer: New Approaches toward Diagnosis and Treatment. Radiology.

[9] Sung H., Ferlay J., Siegel R.L., Laversanne M., Soerjomataram I., Jemal A., Bray F. (2021). Global cancer statistics 2020: GLOBOCAN estimates of incidence and mortality worldwide for 36 cancers in 185 countries. CA Cancer J. Clin..

[10] Tan W., Yang M., Yang H., Zhou F., Shen W. (2018). Predicting the response to neoadjuvant therapy for early-stage breast cancer: Tumor-, blood-, and imaging-related biomarkers. Cancer Manag. Res..

[11] Tirada N., Aujero M., Khorjekar G., Richards S., Chopra J., Dromi S., Ioffe O. (2018). Breast Cancer Tissue Markers, Genomic Profiling, and Other Prognostic Factors: A Primer for Radiologists. Radiographics.

[12] Gradishar W.J., Anderson B.O., Balassanian R., Blair S.L., Burstein H.J., Cyr A., Elias A.D., Farrar W.B., Forero A., Giordano S.H. (2018). Breast Cancer, Version 4.2017, NCCN Clinical Practice Guidelines in Oncology. J. Natl. Compr. Cancer Netw..

[13] Prud’homme C., Deschamps F., Allorant A., Massard C., Hollebecque A., Yevich S., Ngo-Camus M., Gravel G., Nicotra C., Michiels S. (2018). Image-guided tumour biopsies in a prospective molecular triage study (MOSCATO-01): What are the real risks?. Eur. J. Cancer.

[14] Dercle L., Ammari S., Bateson M., Durand P.B., Haspinger E., Massard C., Jaudet C., Varga A., Deutsch E., Soria J.C. (2017). Limits of radiomic-based entropy as a surrogate of tumor heterogeneity: ROI-area, acquisition protocol and tissue site exert substantial influence. Sci. Rep..

[15] Burrell R.A., McGranahan N., Bartek J., Swanton C. (2013). The causes and consequences of genetic heterogeneity in cancer evolution. Nature.

[16] Tselikas L., Sun R., Ammari S., Dercle L., Yevich S., Hollebecque A., Ngo-Camus M., Nicotra C., Deutsch E., Deschamps F. (2019). Role of image-guided biopsy and radiomics in the age of precision medicine. Chin. Clin. Oncol..

[17] Zwanenburg A., Vallieres M., Abdalah M.A., Aerts H., Andrearczyk V., Apte A., Ashrafinia S., Bakas S., Beukinga R.J., Boellaard R. (2020). The Image Biomarker Standardization Initiative: Standardized Quantitative Radiomics for High-Throughput Image-based Phenotyping. Radiology.

[18] Onitilo A.A., Engel J.M., Greenlee R.T., Mukesh B.N. (2009). Breast cancer subtypes based on ER/PR and Her2 expression: Comparison of clinicopathologic features and survival. Clin. Med. Res..

[19] Bundred N.J. (2001). Prognostic and predictive factors in breast cancer. Cancer Treat. Rev..

[20] Curigliano G., Burstein H.J., Winer E.P., Gnant M., Dubsky P., Loibl S., Colleoni M., Regan M.M., Piccart-Gebhart M., Senn H.J. (2019). De-escalating and escalating treatments for early-stage breast cancer: The St. Gallen International Expert Consensus Conference on the Primary Therapy of Early Breast Cancer 2017. Ann. Oncol..

[21] Viale G., Hanlon Newell A.E., Walker E., Harlow G., Bai I., Russo L., Dell’Orto P., Maisonneuve P. (2019). Ki-67 (30-9) scoring and differentiation of Luminal A- and Luminal B-like breast cancer subtypes. Breast Cancer Res. Treat..

[22] Cain E.H., Saha A., Harowicz M.R., Marks J.R., Marcom P.K., Mazurowski M.A. (2018). Multivariate machine learning models for prediction of pathologic response to neoadjuvant therapy in breast cancer using MRI features: A study using an independent validation set. Breast Cancer Res. Treat..

[23] Rotili A., Trimboli R.M., Penco S., Pesapane F., Tantrige P., Cassano E., Sardanelli F. (2020). Double reading of diffusion-weighted magnetic resonance imaging for breast cancer detection. Breast Cancer Res. Treat..

[24] Davnall F., Yip C.S., Ljungqvist G., Selmi M., Ng F., Sanghera B., Ganeshan B., Miles K.A., Cook G.J., Goh V. (2012). Assessment of tumor heterogeneity: An emerging imaging tool for clinical practice?. Insights Imaging.

[25] Yip S.S.F., Parmar C., Kim J., Huynh E., Mak R.H., Aerts H. (2017). Impact of experimental design on PET radiomics in predicting somatic mutation status. Eur. J. Radiol..

[26] Aerts H.J., Velazquez E.R., Leijenaar R.T., Parmar C., Grossmann P., Carvalho S., Bussink J., Monshouwer R., Haibe-Kains B., Rietveld D. (2014). Decoding tumour phenotype by noninvasive imaging using a quantitative radiomics approach. Nat. Commun..

[27] Rahmim A., Salimpour Y., Jain S., Blinder S.A., Klyuzhin I.S., Smith G.S., Mari Z., Sossi V. (2016). Application of texture analysis to DAT SPECT imaging: Relationship to clinical assessments. Neuroimage Clin..

[28] Pesapane F., Patella F., Fumarola E.M., Panella S., Ierardi A.M., Pompili G.G., Franceschelli G., Angileri S.A., Magenta Biasina A., Carrafiello G. (2017). Intravoxel Incoherent Motion (IVIM) Diffusion Weighted Imaging (DWI) in the Periferic Prostate Cancer Detection and Stratification. Med. Oncol..

[29] Patella F., Franceschelli G., Petrillo M., Sansone M., Fusco R., Pesapane F., Pompili G., Ierardi A.M., Saibene A.M., Moneghini L. (2018). A multiparametric analysis combining DCE-MRI- and IVIM-derived parameters to improve differentiation of parotid tumors: A pilot study. Future Oncol..

[30] King A.D., Chow K.K., Yu K.H., Mo F.K., Yeung D.K., Yuan J., Bhatia K.S., Vlantis A.C., Ahuja A.T. (2013). Head and neck squamous cell carcinoma: Diagnostic performance of diffusion-weighted MR imaging for the prediction of treatment response. Radiology.

[31] Peng S.L., Chen C.F., Liu H.L., Lui C.C., Huang Y.J., Lee T.H., Chang C.C., Wang F.N. (2013). Analysis of parametric histogram from dynamic contrast-enhanced MRI: Application in evaluating brain tumor response to radiotherapy. NMR Biomed..

[32] Aerts H.J. (2016). The Potential of Radiomic-Based Phenotyping in Precision Medicine: A Review. JAMA Oncol..

[33] Lee S.H., Park H., Ko E.S. (2020). Radiomics in Breast Imaging from Techniques to Clinical Applications: A Review. Korean J. Radiol..

[34] Bianchini L., Santinha J., Loucao N., Figueiredo M., Botta F., Origgi D., Cremonesi M., Cassano E., Papanikolaou N., Lascialfari A. (2020). A multicenter study on radiomic features from T2-weighted images of a customized MR pelvic phantom setting the basis for robust radiomic models in clinics. Magn. Reson. Med..

[35] Mao N., Yin P., Li Q., Wang Q., Liu M., Ma H., Dong J., Che K., Wang Z., Duan S. (2020). Radiomics nomogram of contrast-enhanced spectral mammography for prediction of axillary lymph node metastasis in breast cancer: A multicenter study. Eur. Radiol..

[36] Hao W., Gong J., Wang S., Zhu H., Zhao B., Peng W. (2020). Application of MRI Radiomics-Based Machine Learning Model to Improve Contralateral BI-RADS 4 Lesion Assessment. Front. Oncol..

[37] Lo Gullo R., Daimiel I., Rossi Saccarelli C., Bitencourt A., Gibbs P., Fox M.J., Thakur S.B., Martinez D.F., Jochelson M.S., Morris E.A. (2020). Improved characterization of sub-centimeter enhancing breast masses on MRI with radiomics and machine learning in BRCA mutation carriers. Eur. Radiol..

[38] Demircioglu A., Grueneisen J., Ingenwerth M., Hoffmann O., Pinker-Domenig K., Morris E., Haubold J., Forsting M., Nensa F., Umutlu L. (2020). A rapid volume of interest-based approach of radiomics analysis of breast MRI for tumor decoding and phenotyping of breast cancer. PLoS ONE.

[39] Zhang Q., Peng Y., Liu W., Bai J., Zheng J., Yang X., Zhou L. (2020). Radiomics Based on Multimodal MRI for the Differential Diagnosis of Benign and Malignant Breast Lesions. J. Magn. Reson. Imaging.

[40] Liu Z., Feng B., Li C., Chen Y., Chen Q., Li X., Guan J., Chen X., Cui E., Li R. (2019). Preoperative prediction of lymphovascular invasion in invasive breast cancer with dynamic contrast-enhanced-MRI-based radiomics. J. Magn. Reson. Imaging.

[41] Xie T., Wang Z., Zhao Q., Bai Q., Zhou X., Gu Y., Peng W., Wang H. (2019). Machine Learning-Based Analysis of MR Multiparametric Radiomics for the Subtype Classification of Breast Cancer. Front. Oncol..

[42] Liang C., Cheng Z., Huang Y., He L., Chen X., Ma Z., Huang X., Liang C., Liu Z. (2018). An MRI-based Radiomics Classifier for Preoperative Prediction of Ki-67 Status in Breast Cancer. Acad. Radiol..

[43] Tan H., Wu Y., Bao F., Zhou J., Wan J., Tian J., Lin Y., Wang M. (2020). Mammography-based radiomics nomogram: A potential biomarker to predict axillary lymph node metastasis in breast cancer. Br. J. Radiol..

[44] Stelzer P.D., Steding O., Raudner M.W., Euller G., Clauser P., Baltzer P.A.T. (2020). Combined texture analysis and machine learning in suspicious calcifications detected by mammography: Potential to avoid unnecessary stereotactical biopsies. Eur. J. Radiol..

[45] Lin F., Wang Z., Zhang K., Yang P., Ma H., Shi Y., Liu M., Wang Q., Cui J., Mao N. (2020). Contrast-Enhanced Spectral Mammography-Based Radiomics Nomogram for Identifying Benign and Malignant Breast Lesions of Sub-1 cm. Front. Oncol..

[46] Zhou J., Tan H., Bai Y., Li J., Lu Q., Chen R., Zhang M., Feng Q., Wang M. (2019). Evaluating the HER-2 status of breast cancer using mammography radiomics features. Eur. J. Radiol..

[47] Lei C., Wei W., Liu Z., Xiong Q., Yang C., Yang M., Zhang L., Zhu T., Zhuang X., Liu C. (2019). Mammography-based radiomic analysis for predicting benign BI-RADS category 4 calcifications. Eur. J. Radiol..

[48] Ou X., Zhang J., Wang J., Pang F., Wang Y., Wei X., Ma X. (2019). Radiomics based on (18) F-FDG PET/CT could differentiate breast carcinoma from breast lymphoma using machine-learning approach: A preliminary study. Cancer Med..

[49] Zhou J., Zhang Y., Chang K.T., Lee K.E., Wang O., Li J., Lin Y., Pan Z., Chang P., Chow D. (2019). Diagnosis of Benign and Malignant Breast Lesions on DCE-MRI by Using Radiomics and Deep Learning With Consideration of Peritumor Tissue. J. Magn. Reson. Imaging.

[50] Reig B., Heacock L., Geras K.J., Moy L. (2020). Machine learning in breast MRI. J. Magn. Reson. Imaging.

[51] Granzier R.W.Y., van Nijnatten T.J.A., Woodruff H.C., Smidt M.L., Lobbes M.B.I. (2019). Exploring breast cancer response prediction to neoadjuvant systemic therapy using MRI-based radiomics: A systematic review. Eur. J. Radiol..

[52] American Cancer Society Breast Cancer Survival Rates by Stage. http://www.cancer.org/cancer/breastcancer/detailedguide/breast-cancer-survival-by-stage.

[53] NCCN.org (2018). Breast Cancer Screening and Diagnosis Version 3. NCCN Clinical Practice Guidelines in Oncology (NCCN Guidelines).

[54] Mann R.M., Balleyguier C., Baltzer P.A., Bick U., Colin C., Cornford E., Evans A., Fallenberg E., Forrai G., Fuchsjager M.H. (2015). Breast MRI: EUSOBI recommendations for women’s information. Eur. Radiol..

[55] Jiang X., Xie F., Liu L., Peng Y., Cai H., Li L. (2018). Discrimination of malignant and benign breast masses using automatic segmentation and features extracted from dynamic contrast-enhanced and diffusion-weighted MRI. Oncol. Lett..

[56] Bickelhaupt S., Jaeger P.F., Laun F.B., Lederer W., Daniel H., Kuder T.A., Wuesthof L., Paech D., Bonekamp D., Radbruch A. (2018). Radiomics Based on Adapted Diffusion Kurtosis Imaging Helps to Clarify Most Mammographic Findings Suspicious for Cancer. Radiology.

[57] Li H., Mendel K.R., Lan L., Sheth D., Giger M.L. (2019). Digital Mammography in Breast Cancer: Additive Value of Radiomics of Breast Parenchyma. Radiology.

[58] Tagliafico A.S., Valdora F., Mariscotti G., Durando M., Nori J., La Forgia D., Rosenberg I., Caumo F., Gandolfo N., Houssami N. (2018). An exploratory radiomics analysis on digital breast tomosynthesis in women with mammographically negative dense breasts. Breast.

[59] Massafra R., Bove S., Lorusso V., Biafora A., Comes M.C., Didonna V., Diotaiuti S., Fanizzi A., Nardone A., Nolasco A. (2021). Radiomic Feature Reduction Approach to Predict Breast Cancer by Contrast-Enhanced Spectral Mammography Images. Diagnostics.

[60] Luo W.Q., Huang Q.X., Huang X.W., Hu H.T., Zeng F.Q., Wang W. (2019). Predicting Breast Cancer in Breast Imaging Reporting and Data System (BI-RADS) Ultrasound Category 4 or 5 Lesions: A Nomogram Combining Radiomics and BI-RADS. Sci. Rep..

[61] Fan M., Li H., Wang S., Zheng B., Zhang J., Li L. (2017). Radiomic analysis reveals DCE-MRI features for prediction of molecular subtypes of breast cancer. PLoS ONE.

[62] Xie T., Zhao Q., Fu C., Bai Q., Zhou X., Li L., Grimm R., Liu L., Gu Y., Peng W. (2019). Differentiation of triple-negative breast cancer from other subtypes through whole-tumor histogram analysis on multiparametric MR imaging. Eur. Radiol..

[63] La Forgia D., Fanizzi A., Campobasso F., Bellotti R., Didonna V., Lorusso V., Moschetta M., Massafra R., Tamborra P., Tangaro S. (2020). Radiomic Analysis in Contrast-Enhanced Spectral Mammography for Predicting Breast Cancer Histological Outcome. Diagnostics.

[64] Early Breast Cancer Trialists’ Collaborative Group (EBCTCG) (2018). Long-term outcomes for neoadjuvant versus adjuvant chemotherapy in early breast cancer: Meta-analysis of individual patient data from ten randomised trials. Lancet Oncol..

[65] Tyagi N.K., Dhesy-Thind S. (2018). Clinical practice guidelines in breast cancer. Curr. Oncol..

[66] Krop I., Ismaila N., Andre F., Bast R.C., Barlow W., Collyar D.E., Hammond M.E., Kuderer N.M., Liu M.C., Mennel R.G. (2017). Use of Biomarkers to Guide Decisions on Adjuvant Systemic Therapy for Women With Early-Stage Invasive Breast Cancer: American Society of Clinical Oncology Clinical Practice Guideline Focused Update. J. Clin. Oncol..

[67] Liu Z., Li Z., Qu J., Zhang R., Zhou X., Li L., Sun K., Tang Z., Jiang H., Li H. (2019). Radiomics of Multiparametric MRI for Pretreatment Prediction of Pathologic Complete Response to Neoadjuvant Chemotherapy in Breast Cancer: A Multicenter Study. Clin. Cancer Res..

[68] Xiong Q., Zhou X., Liu Z., Lei C., Yang C., Yang M., Zhang L., Zhu T., Zhuang X., Liang C. (2019). Multiparametric MRI-based radiomics analysis for prediction of breast cancers insensitive to neoadjuvant chemotherapy. Clin. Transl. Oncol..

[69] Drukker K., Li H., Antropova N., Edwards A., Papaioannou J., Giger M.L. (2018). Most-enhancing tumor volume by MRI radiomics predicts recurrence-free survival "early on" in neoadjuvant treatment of breast cancer. Cancer Imaging.

[70] Teruel J.R., Heldahl M.G., Goa P.E., Pickles M., Lundgren S., Bathen T.F., Gibbs P. (2014). Dynamic contrast-enhanced MRI texture analysis for pretreatment prediction of clinical and pathological response to neoadjuvant chemotherapy in patients with locally advanced breast cancer. NMR Biomed..

[71] Braman N.M., Etesami M., Prasanna P., Dubchuk C., Gilmore H., Tiwari P., Plecha D., Madabhushi A. (2017). Intratumoral and peritumoral radiomics for the pretreatment prediction of pathological complete response to neoadjuvant chemotherapy based on breast DCE-MRI. Breast Cancer Res..

[72] Fan M., Wu G., Cheng H., Zhang J., Shao G., Li L. (2017). Radiomic analysis of DCE-MRI for prediction of response to neoadjuvant chemotherapy in breast cancer patients. Eur. J. Radiol..

[73] Ahmed A., Gibbs P., Pickles M., Turnbull L. (2013). Texture analysis in assessment and prediction of chemotherapy response in breast cancer. J. Magn. Reson. Imaging.

[74] Parikh J., Selmi M., Charles-Edwards G., Glendenning J., Ganeshan B., Verma H., Mansi J., Harries M., Tutt A., Goh V. (2014). Changes in primary breast cancer heterogeneity may augment midtreatment MR imaging assessment of response to neoadjuvant chemotherapy. Radiology.

[75] Wang Z., Lin F., Ma H., Shi Y., Dong J., Yang P., Zhang K., Guo N., Zhang R., Cui J. (2021). Contrast-Enhanced Spectral Mammography-Based Radiomics Nomogram for the Prediction of Neoadjuvant Chemotherapy-Insensitive Breast Cancers. Front. Oncol..

[76] Galimberti V., Cole B.F., Viale G., Veronesi P., Vicini E., Intra M., Mazzarol G., Massarut S., Zgajnar J., Taffurelli M. (2018). Axillary dissection versus no axillary dissection in patients with breast cancer and sentinel-node micrometastases (IBCSG 23-01): 10-year follow-up of a randomised, controlled phase 3 trial. Lancet Oncol..

[77] Galimberti V., Cole B.F., Zurrida S., Viale G., Luini A., Veronesi P., Baratella P., Chifu C., Sargenti M., Intra M. (2013). Axillary dissection versus no axillary dissection in patients with sentinel-node micrometastases (IBCSG 23-01): A phase 3 randomised controlled trial. Lancet Oncol..

[78] Veronesi U., Paganelli G., Viale G., Luini A., Zurrida S., Galimberti V., Intra M., Veronesi P., Maisonneuve P., Gatti G. (2006). Sentinel-lymph-node biopsy as a staging procedure in breast cancer: Update of a randomised controlled study. Lancet Oncol..

[79] Charalampoudis P., Markopoulos C., Kovacs T. (2018). Controversies and recommendations regarding sentinel lymph node biopsy in primary breast cancer: A comprehensive review of current data. Eur. J. Surg. Oncol..

[80] Dihge L., Bendahl P.O., Ryden L. (2017). Nomograms for preoperative prediction of axillary nodal status in breast cancer. Br. J. Surg..

[81] Choi E.J., Youk J.H., Choi H., Song J.S. (2020). Dynamic contrast-enhanced and diffusion-weighted MRI of invasive breast cancer for the prediction of sentinel lymph node status. J. Magn. Reson. Imaging.

[82] Dong Y., Feng Q., Yang W., Lu Z., Deng C., Zhang L., Lian Z., Liu J., Luo X., Pei S. (2018). Preoperative prediction of sentinel lymph node metastasis in breast cancer based on radiomics of T2-weighted fat-suppression and diffusion-weighted MRI. Eur. Radiol..

[83] Han L., Zhu Y., Liu Z., Yu T., He C., Jiang W., Kan Y., Dong D., Tian J., Luo Y. (2019). Radiomic nomogram for prediction of axillary lymph node metastasis in breast cancer. Eur. Radiol..

[84] Cui X., Wang N., Zhao Y., Chen S., Li S., Xu M., Chai R. (2019). Preoperative Prediction of Axillary Lymph Node Metastasis in Breast Cancer using Radiomics Features of DCE-MRI. Sci. Rep..

[85] Liu C., Ding J., Spuhler K., Gao Y., Serrano Sosa M., Moriarty M., Hussain S., He X., Liang C., Huang C. (2019). Preoperative prediction of sentinel lymph node metastasis in breast cancer by radiomic signatures from dynamic contrast-enhanced MRI. J. Magn. Reson. Imaging.

[86] Yang J., Wang T., Yang L., Wang Y., Li H., Zhou X., Zhao W., Ren J., Li X., Tian J. (2019). Preoperative Prediction of Axillary Lymph Node Metastasis in Breast Cancer Using Mammography-Based Radiomics Method. Sci. Rep..

[87] Yu F.H., Wang J.X., Ye X.H., Deng J., Hang J., Yang B. (2019). Ultrasound-based radiomics nomogram: A potential biomarker to predict axillary lymph node metastasis in early-stage invasive breast cancer. Eur. J. Radiol..

[88] Yang X., Wu L., Ye W., Zhao K., Wang Y., Liu W., Li J., Li H., Liu Z., Liang C. (2020). Deep Learning Signature Based on Staging CT for Preoperative Prediction of Sentinel Lymph Node Metastasis in Breast Cancer. Acad. Radiol..

[89] Ye D.M., Wang H.T., Yu T. (2020). The Application of Radiomics in Breast MRI: A Review. Technol. Cancer Res. Treat..

[90] Da-Ano R., Masson I., Lucia F., Dore M., Robin P., Alfieri J., Rousseau C., Mervoyer A., Reinhold C., Castelli J. (2020). Performance comparison of modified ComBat for harmonization of radiomic features for multicenter studies. Sci. Rep..

[91] Chaudhary K., Poirion O.B., Lu L., Garmire L.X. (2018). Deep Learning-Based Multi-Omics Integration Robustly Predicts Survival in Liver Cancer. Clin. Cancer Res..

[92] Abajian A., Murali N., Savic L.J., Laage-Gaupp F.M., Nezami N., Duncan J.S., Schlachter T., Lin M., Geschwind J.F., Chapiro J. (2018). Predicting Treatment Response to Intra-arterial Therapies for Hepatocellular Carcinoma with the Use of Supervised Machine Learning-An Artificial Intelligence Concept. J. Vasc. Interv. Radiol..

[93] El-Sayed M.E., Rakha E.A., Reed J., Lee A.H., Evans A.J., Ellis I.O. (2008). Predictive value of needle core biopsy diagnoses of lesions of uncertain malignant potential (B3) in abnormalities detected by mammographic screening. Histopathology.

[94] Miller D.D., Brown E.W. (2018). Artificial Intelligence in Medical Practice: The Question to the Answer?. Am. J. Med..

[95] Samuel A.L. (1959). Some studies in machine learning using the game of checkers. IBM J. Res. Dev..

[96] Lee J.G., Jun S., Cho Y.W., Lee H., Kim G.B., Seo J.B., Kim N. (2017). Deep Learning in Medical Imaging: General Overview. Korean J. Radiol..

[97] LeCun Y., Bengio Y., Hinton G. (2015). Deep learning. Nature.

[98] Thrall J.H., Li X., Li Q., Cruz C., Do S., Dreyer K., Brink J. (2018). Artificial Intelligence and Machine Learning in Radiology: Opportunities, Challenges, Pitfalls, and Criteria for Success. J. Am. Coll. Radiol..

[99] Pesapane F., Volonte C., Codari M., Sardanelli F. (2018). Artificial intelligence as a medical device in radiology: Ethical and regulatory issues in Europe and the United States. Insights Imaging.

[100] Nie D., Trullo R., Lian J., Wang L., Petitjean C., Ruan S., Wang Q., Shen D. (2018). Medical Image Synthesis with Deep Convolutional Adversarial Networks. IEEE Trans. Biomed. Eng..

[101] Havaei M., Davy A., Warde-Farley D., Biard A., Courville A., Bengio Y., Pal C., Jodoin P.M., Larochelle H. (2017). Brain tumor segmentation with Deep Neural Networks. Med. Image Anal..

[102] Mayer-Schonberger V., Ingelsson E. (2018). Big Data and medicine: A big deal?. J. Intern. Med..

[103] Yi P.H., Hui F.K., Ting D.S.W. (2018). Artificial Intelligence and Radiology: Collaboration Is Key. J. Am. Coll. Radiol..

[104] Swensen S.J., Johnson C.D. (2005). Radiologic quality and safety: Mapping value into radiology. J. Am. Coll. Radiol..

[105] Pesapane F. (2019). How scientific mobility can help current and future radiology research: A radiology trainee’s perspective. Insights Imaging.

[106] Kruse C.S., Goswamy R., Raval Y., Marawi S. (2016). Challenges and Opportunities of Big Data in Health Care: A Systematic Review. JMIR Med. Inform..

[107] Larson D.B., Towbin A.J., Pryor R.M., Donnelly L.F. (2013). Improving consistency in radiology reporting through the use of department-wide standardized structured reporting. Radiology.

[108] Schwartz L.H., Panicek D.M., Berk A.R., Li Y., Hricak H. (2011). Improving communication of diagnostic radiology findings through structured reporting. Radiology.

[109] Pereira S., Pinto A., Alves V., Silva C.A. (2016). Brain Tumor Segmentation Using Convolutional Neural Networks in MRI Images. IEEE Trans. Med. Imaging.

[110] Moeskops P., Viergever M.A., Mendrik A.M., de Vries L.S., Benders M.J., Isgum I. (2016). Automatic Segmentation of MR Brain Images With a Convolutional Neural Network. IEEE Trans. Med. Imaging.

[111] Kruskal J.B., Berkowitz S., Geis J.R., Kim W., Nagy P., Dreyer K. (2017). Big Data and Machine Learning-Strategies for Driving This Bus: A Summary of the 2016 Intersociety Summer Conference. J. Am. Coll. Radiol..

[112] Kansagra A.P., Yu J.P., Chatterjee A.R., Lenchik L., Chow D.S., Prater A.B., Yeh J., Doshi A.M., Hawkins C.M., Heilbrun M.E. (2016). Big Data and the Future of Radiology Informatics. Acad. Radiol..

[113] Ranschaert E.R., Sergey M., Algra P.R. (2019). Artificial Intelligence in Medical Imaging.

[114] Krittanawong C. (2018). The rise of artificial intelligence and the uncertain future for physicians. Eur. J. Intern. Med..

[115] Collins G.S., Moons K.G.M. (2019). Reporting of artificial intelligence prediction models. Lancet.

[116] Lambin P., Leijenaar R.T.H., Deist T.M., Peerlings J., de Jong E.E.C., van Timmeren J., Sanduleanu S., Larue R., Even A.J.G., Jochems A. (2017). Radiomics: The bridge between medical imaging and personalized medicine. Nat. Rev. Clin. Oncol..

[117] Sanduleanu S., Woodruff H.C., de Jong E.E.C., van Timmeren J.E., Jochems A., Dubois L., Lambin P. (2018). Tracking tumor biology with radiomics: A systematic review utilizing a radiomics quality score. Radiother. Oncol..

[118] Collins G.S., Reitsma J.B., Altman D.G., Moons K.G. (2015). Transparent Reporting of a multivariable prediction model for Individual Prognosis or Diagnosis (TRIPOD): The TRIPOD statement. Ann. Intern. Med..

[119] Kemp J.L., Mahoney M.C., Mathews V.P., Wintermark M., Yee J., Brown S.D. (2017). Patient-centered Radiology: Where Are We, Where Do We Want to Be, and How Do We Get There?. Radiology.

[120] Castelvecchi D. (2016). Can we open the black box of AI?. Nature.

[121] Chevrier R., Foufi V., Gaudet-Blavignac C., Robert A., Lovis C. (2019). Use and Understanding of Anonymization and De-Identification in the Biomedical Literature: Scoping Review. J. Med. Internet Res..

[122] The European Parliament and the Council of The European Union Directive (EU) 2016/1148 of the European Parliament and of the Council Concerning Measures for a High Common Level of Security of Network and Information Systems across the Union. https://eur-lex.europa.eu/legal-content/EN/TXT/?toc=OJ:L:2016:194:TOC&uri=uriserv:OJ.L_.2016.194.01.0001.01.ENG.

[123] Tsang L., Kracov D.A., Mulryne J., Strom L., Perkins N., Dickinson R., Wallace V.M., Jones B. The Impact of Artificial Intelligence on Medical Innovation in the European Union and United States. https://www.arnoldporter.com/~/media/files/perspectives/publications/2017/08/the-impact-of-artificial-inteelligence-on-medical-innovation.pdf.

[124] Jiang F., Jiang Y., Zhi H., Dong Y., Li H., Ma S., Wang Y., Dong Q., Shen H., Wang Y. (2017). Artificial intelligence in healthcare: Past, present and future. Stroke Vasc. Neurol..

[125] The Cancer Imaging Archive (TCIA). http://www.cancerimagingarchive.net.

[126] Fonseca C.G., Backhaus M., Bluemke D.A., Britten R.D., Chung J.D., Cowan B.R., Dinov I.D., Finn J.P., Hunter P.J., Kadish A.H. (2011). The Cardiac Atlas Project—An imaging database for computational modeling and statistical atlases of the heart. Bioinformatics.

[127] Jimenez-Del-Toro O., Muller H., Krenn M., Gruenberg K., Taha A.A., Winterstein M., Eggel I., Foncubierta-Rodriguez A., Goksel O., Jakab A. (2016). Cloud-Based Evaluation of Anatomical Structure Segmentation and Landmark Detection Algorithms: VISCERAL Anatomy Benchmarks. IEEE Trans. Med. Imaging.

[128] UK. http://www.ukbiobank.ac.uk/.

[129] He J., Baxter S.L., Xu J., Xu J., Zhou X., Zhang K. (2019). The practical implementation of artificial intelligence technologies in medicine. Nat. Med..

[130] Hashimoto D.A., Rosman G., Rus D., Meireles O.R. (2018). Artificial Intelligence in Surgery: Promises and Perils. Ann. Surg..

